# Measuring factors that influence the utilisation of preventive care services provided by general practitioners in Australia

**DOI:** 10.1186/1472-6963-9-218

**Published:** 2009-12-03

**Authors:** Jianzhen Zhang, Brian Oldenburg, Gavin Turrell

**Affiliations:** 1 School of Medicine, University of Queensland, Herston Road, Herston, Brisbane QLD 4006, Australia; 2 School of Public Health and Preventive Medicine, Monash University, Victoria, Australia; 3 School of Public Health, Queensland University of Technology, Queensland, Australia

## Abstract

**Background:**

Relatively little research attention has been given to the development of standardised and psychometrically sound scales for measuring influences relevant to the utilisation of health services. This study aims to describe the development, validation and internal reliability of some existing and new scales to measure factors that are likely to influence utilisation of preventive care services provided by general practitioners in Australia.

**Methods:**

Relevant domains of influence were first identified from a literature review and formative research. Items were then generated by using and adapting previously developed scales and published findings from these. The new items and scales were pre-tested and qualitative feedback was obtained from a convenience sample of citizens from the community and a panel of experts. Principal Components Analyses (PCA) and internal reliability testing (Cronbach's *alpha*) were then conducted for all of the newly adapted or developed scales utilising data collected from a self-administered mailed survey sent to a randomly selected population-based sample of 381 individuals (response rate 65.6 per cent).

**Results:**

The PCA identified five scales with acceptable levels of internal consistency were: (1) social support (ten items), *alpha *0.86; (2) perceived interpersonal care (five items), *alpha *0.87, (3) concerns about availability of health care and accessibility to health care (eight items), *alpha *0.80, (4) value of good health (five items), *alpha *0.79, and (5) attitudes towards health care (three items), *alpha *0.75.

**Conclusion:**

The five scales are suitable for further development and more widespread use in research aimed at understanding the determinants of preventive health services utilisation among adults in the general population.

## Background

Considerable international research has examined the factors that influence people's use of preventive health services delivered by health professionals such as general practitioners (GPs). This research demonstrates that many of the most salient factors occur not just at the individual level, but also at the interpersonal, organisational/environmental and system levels [1-6]. At the individual level, patients' attitudes and beliefs have been clearly demonstrated to be related to their access and use of preventive health services provided by GPs and various kinds of clinics. For example, belief in regular physical check-ups was positively associated with self-reported utilisation of outpatient medical care [7]. Studies in the US and UK found that a lack of appropriate knowledge and understanding about screening mammography were key factors in women not seeking out screening [8,9].

At the interpersonal or social level, factors can be classified into two groups that include family resources such as health insurance coverage and accessibility of a regular source of health care, and social support from family, friends and other social groups. For example, people with a regular source of care and appropriate medical insurance are more likely to have a routine medical check-up than those without a regular source of care or medical insurance [10-15]. A number of Australian studies have also suggested that patients who utilise the same GPs on repeat occasions are more likely to receive appropriate preventive care [16,17]. Different US studies have demonstrated the importance of social network variables in accounting for differences in the use of preventive health services such as medical check-ups, dental care and immunisations [18,19]. In addition, Australian studies suggest that family and social support are important influences on people's use of preventive health services. For example, men with lower social support were less likely to contact a GP [20].

At the environmental/organisational level, access to a car, short distance and travel time to health services and waiting time in health services are positive determinants of preventive health utilisation [8,21,22]. Finally, at the system level, health services utilisation behaviour is also strongly influenced by societal resources, priorities and policies. For example, in Australia, the Medicare rebate system supports (almost) universal access to GP services compared to other countries such as the US [23]; however, even in this system, the majority of GPs still require a co-payment to be paid by their patients and there are also other (in)direct costs associated with visiting a GP [24,25]. Young et al. [25] found that lower socioeconomic position was associated with lower out-of-pocket costs per consultation and was inversely related to use of preventive health services.

Andersen's Behavioral Model of Health Services Utilisation has been widely used for many years as a theoretical model for understanding individuals' use of health services (including preventive health services) [26]. This model was initially developed and empirically tested in a series of studies to help understand why families use health services and also to define and measure equitable access to health care and to assist in developing policies to promote equitable access. The model has been updated a number of times since the 1960's [1,4]. It purports to identify the multiple factors that influence health services use [1]. The theoretical framework used for the present study was based primarily on Andersen's Behavioural Model of Health Services Utilisation, but also included some additional elements from other models (such as the Health Belief Model). It focused on four levels of factors, with these being at the system, organisational/environmental, interpersonal and individual levels of influence, with a particular focus on those factors which might best predict socioeconomic differences in use of preventive health services. This adapted model was used in the current study to inform the further development and psychometric evaluation of measures of those key influences of health services related to the aforementioned four levels, including: perceived availability of and accessibility to health care; perceived interpersonal care from GPs; regular source of care; social support; and attitudes and beliefs towards health care and GPs. Given that relatively little attention has been given to the development of valid and reliable scales for measuring these kinds of influences, the current study aims to describe the development, validation and internal reliability of some existing and new scales to measure factors that are likely to influence utilisation of preventive care services provided by general practitioners in Australia.

## Methods

### Scale Development

The scales and questionnaire were developed according to the following steps: (1) identification of relevant and salient domains; (2) identification and/or adaptation of existing items and where necessary, development of new items; (3) development of a draft questionnaire and review by an expert panel; (4) pre-testing and piloting of the reviewed questionnaire; (5) collection of data using a mail survey; and (6) evaluation of the psychometric properties of the scales. Each of these critical steps and the results are discussed in the following section.

#### Identification of relevant and salient domains

In order to identify relevant domains of influences on GP preventive health services utilisation, a literature review was conducted. The key search terms used were: 'primary health care' or 'general practice', 'prevention' or 'preventive activities', 'preventive health services or preventive health care', and determinants of health services utilisation'. The search was conducted via the EBSCO HOST search engine, including Medline, PsychINFO, Primary Search and PsycARTICLES databases. The most commonly investigated determinants of GP preventive health services utilisation have been: (1) the type of health care system as a system-level determinant, such as bulk-billing services; (2) environmental/organisational-level factors affecting access, including transport, travel time and cost; the number of doctors or health professionals to choose from, being able to see a preferred doctor, appointment time, waiting time and length of consultation time; (3) interpersonal-level factors such as family sources and patient social support variables; and finally, (4) the individual's attitudes and beliefs towards different aspects of health and health care. Table 1 summarises these four domains and gives examples of the kinds concepts relevant to each domain.

**Table 1:** Domains of influences on GP preventive health services utilisation

Domains	Details	Description
**(1) Perceived system-level factor**		
Health care system	Being able to find a doctor who	For example, bulk billing is the Medicare rebate system in Australia, which allows GPs to provide their services at no 'out-of-pocket' cost to patients
	bulk bills	
**(2) Perceived environmental/organisational-level factors**		
Accessibility to health services	Accessibility to transport	Transport to GP clinic: public/own car to access to health services.
	Travel time to health services	Travel time to the regular source of medical care.
	The cost of seeing a general practitioner	Out-of-pocket cost from patients related to the consultation fee.
Availability of health services	A number of doctors to choose from	Available doctors for patients to choose e.g. female doctors.
	Able to see preferred doctor	A doctor who patients would like to see.
	Amount of time to get an appointment	Elapsed time between initial request and the date of the appointment to see a GP.
	Waiting time	Waiting time in GP's office or clinic.
	Consultation time	How long GP consults with the patient.
GP's attitudes towards patients	Inter-personal care scale	GP's perceived attitude towards patients.

**(3) Interpersonal-level**		
Family sources	Regular source of care	Regularly visit GPs at one practice or one doctor.
Social support	Social support	Support from family, friends and neighbours

**(4) Individual-level**		
Attitudes and beliefs towards health care	Value of general practitioners	Patients' perceptions towards doctors.
	Value of health care	Patients' perceptions towards health care.
	Value of good health	Patients' attitudes towards doctor's advice about improving their health.

#### Identification and/or adaptation of existing items and where necessary, development of new items

Once the domains were identified, a further literature search was conducted in order to identify scales and questionnaire items relevant to each of the domains listed in Table 1. The key search terms used to identify potentially relevant questionnaires, questions and/or scales were, 'preventive health services or preventive health care and beliefs or attitudes or behaviours and instrument or scales'. The search was conducted via the EBSCO HOST search engine, including Medline, PsychINFO, Primary Search and PsycARTICLES databases. The search was also conducted through international and Australian instrument web sites, including the Australian Centre on Quality of Life Instrument Database at http://acqol.deakin.edu.au/instruments/index.htm and BUROS Institute of Mental Measurements at http://www.unl.edu/buros. Key researchers names were also searched for their publications.

Potentially relevant scales and questions relating to each domain were then extracted from a review of all the existing questionnaires and formative studies. The authors of the relevant scales and questions were contacted, and permission was sought to use and/or adapt the previously developed measures. Additional psychometric information and any other relevant information for each of these scales were also requested from the researchers. The criteria we used for determining whether a scale was suitable and appropriate for use in the present study included: the scale had been previously tested psychometrically and validated; the content of the scale was suitable for the new research purpose; and the questionnaire items and permission could be obtained from the author. Subsequently, six scales were identified. They included: (1) concerns about availability of and accessibility to health care [27,28]; (2) perceived interpersonal care [29,30]; (3) value of health care, (4) value of doctors and (5) value of good health [21]; and (6) social support [31-33]. These scales measured people's attitudes and perceptions towards preventive health care and health services at individual, interpersonal, environmental/organisational and system levels.

The items for the scale measuring "concerns about availability and accessibility to health care" were adapted from a previous study [28]. Three items previously used to assess accessibility to health care [27,28] were adapted and re-worded. For example, to obtain more specific information, the original item 'Number of GPs you have to choose from' was changed to 'Having a number of doctors to choose from in the one medical practice/centre'; 'Ease of seeing the GP of your choice' was changed to 'Being able to see my preferred doctor every time'; and 'How long you wait to get a GP appointment' was changed to 'The amount of time it takes to get an appointment to see a doctor'. In order to assess availability of "bulk billing" in general practice, one new item was developed to fit into the scale to assess the availability and accessibility of GP services. Items used previously to assess GP's attitudes towards patients (inter-personal care scale) [29,30] were included in their original form. Items used to measure social support from family and friends [31,32] were slightly reworded such as 'confide in' was changed to 'talk to' and 'come to visit' into 'visit' in order to make the sentence simple and easy understand. Under the domain of individual-level factors, items from previous scales assessing patients' attitudes and beliefs including value of doctors, value of health care and value of good health [15] were reworded based on Australian health system and language. The scale names were changed from "value of doctors" to "value of GPs", from "value of health care" to "Attitudes towards health care", while "value of good health" was left unchanged.

Six scales were developed for further psychometric evaluation. This includes one original scale and five adapted and further developed scales: (1) concerns about availability and accessibility to health care (nine items), (2) perceived interpersonal care (three items) (original scale), (3) attitude towards health care (five items), (4) value of general practitioners (four items) and (5)value of good health (five items), and (6)social support (ten items). Items of concerns about availability and accessibility to health care used a six-point response format with responses including 'very important', 'fairly important', 'not important', 'not at all important', 'I don't think about it' and 'not applicable'. Items of perceived interpersonal care used a five-point response format comprising 'excellent', 'very good', 'good', 'fair' and 'poor'. Items of attitude towards health care, value of general practitioners and social support used a seven-point response format with responses ranging from 'strongly agree' to 'strongly disagree'. Finally, items concerning the value of good health used four-point response format with responses ranging from 'very likely' to 'very unlikely'.

#### Development of a draft questionnaire and review by an expert panel

The items for the 6 scales (36 items) were then combined together with 12 socio-demographic items, 10 items assessing health status, disease conditions and smoking status, 20 items assessing use of health services and finally one item assessing regular source of health care. Combined together, the items from the 6 scales and all the additional items formed the GP Preventive Health Services Utilisation Questionnaire (GP-PHSUQ). This draft questionnaire was then reviewed by six "expert" national and international researchers working in one or more of the fields of health services, primary health care or public health research. The experts were requested to consider two main issues: first, whether the proposed scales and questions provided sufficient coverage of the required domain areas; and, secondly, whether the expert was aware of other existing measures or questionnaires that might further inform item and scale development. Most of the feedback related to specific suggestions about the reading level required to understand some of the questions, formatting of the questionnaire, and the wording and sequencing of questions. Consequently, a number of terms were re-worded so as to reduce the required reading age. A revised version of GP-PHSUQ was developed following the incorporation of the experts' feedback.

#### Pre-testing and piloting of the reviewed questionnaire

The revised questionnaire was then pre-tested with a small, purposive community sample in Brisbane in order to evaluate and further improve the wording of the questions and the presentation of the questionnaire. Twenty two individuals aged from 25 to 64 years, and from different socioeconomic backgrounds were selected for pre-testing. The average age of participants for the pre-testing was 37. Twelve males and ten females participated in this pre-testing. A questionnaire was distributed to each participant, with an accompanying invitation letter and checklist. Feedback and comments provided by participants related to clarity of instructions, repetition of some items, irrelevancy of items, length of questions, similarity of items and sensitivity of questions such as income. Further modification was made to the questionnaire following this feedback.

On completion of the above steps, the final GP-PHSUQ consisted of a 10-page A4 booklet (the questionnaire is available from the first author on request). The questionnaire included 79 items made up of: 12 socio-demographic items; 10 items assessing health status, disease conditions and smoking status; 20 items assessing use of health services; and 37 items assessing the factors that might affect health services utilisation.

#### Collection of data using a mail survey

A mailed survey was then conducted to test reliability and validity of the scales in the general population. A sample of individuals aged 25-64 years of age (N = 800) from Brisbane municipal area was randomly drawn from the Australian Electoral Roll in 2004. The sample included all eligible citizens 18 years of age and over who are registered to vote in Australian elections. The questionnaire was mailed to each selected participant during November 2004. We used Dillman's mail survey methodology [34] as outlined here. Eight hundred surveys were mailed, with each package containing a personalised cover letter, a survey, an instant lottery ticket, as well as a pre-addressed and prepaid reply envelope. After one week, a thank-you and reminder postcard was sent to all participants. A replacement questionnaire with cover letters was sent to non-respondents four weeks after the first questionnaire mail-out. Finally, a reminder letter was sent to those people who had still not returned their survey six weeks after the first mail-out. There were 1453 questionnaires sent out to participants in the three subsequent mailings over six weeks. A final useable response rate of 65.6 per cent was achieved, which included a sample of 519 respondents.

There were 381 respondents' surveys that were eligible to be used for the final data analysis after exclusion of those respondents who already had a pre-existing cardiovascular diseases, diabetes or other self-identified chronic condition (n = 138). The rationale for excluding the latter group from this analysis was that the purpose of this study was to investigate the utilisation of preventive health services by different social economic individuals who did not have any (known) chronic illness at the time of questionnaire completion. There were a total of 381 respondents 25-64 year old, including 155 males (40.7 per cent) and 226 females (59.3 per cent). The average age of the sample was 42. Table 2 summarises the socio-demographic profile of the respondents (N = 381) in terms of their age, gender, education, and household income.

**Table 2:** Socio-demographic characteristics of the study sample (n = 381)

Study variables	No of cases	Percentage
**Gender**		
Male	155	40.7
Female	226	59.3
		
**Age groups**		
25-29	46	12.1
30-34	65	17.1
35-39	61	16.0
40-44	54	14.2
45-49	54	14.2
50-54	40	10.5
55-59	38	10.0
60-64	23	6.0
		
**Education**		
Bachelor degree and higher	151	39.6
Diploma	40	10.5
Vocational	65	17.1
Non post-school qualification	116	30.4
Missing	9	2.4
		
**Household income**		
Aus $52 000 or more	206	54.1
$31 200-51 999	60	15.7
< $31 199	40	10.5
Don't wish to answer	70	18.4
Missing	5	1.3

#### Evaluation of the psychometric properties of the scales

Face and content validity were evaluated for each scale during their development. Face validity was assessed by the research team and the expert review panel. Content validity of the scales and items was evaluated utilising the conceptual framework used for measurement development. Principal Components Analyses (PCA) were conducted in order to extract the maximum amount of variance from the loadings within components across all of the scale items [35]. An initial PCA was performed to determine how many underlying factors were in the complete set of scale items. The number of factors was derived based on eigenvalus greater than 1 and also a scree test of eigenvalues plotted against factors. After the number of factors had been determined, Varimax rotation was used to extract and rotate the final loadings. Items were excluded if the loading of the coefficients was less than 0.5, to allow for a moderate level (20-30 per cent) of overlapping variance [36].

The internal consistency of each factorially derived scale was assessed by calculating Cronbach's *alpha *reliability coefficients for each scale. A Cronbach's *alpha *coefficient of greater than 0.7 was considered acceptable [36]. Mean scores and standard deviations for each factorial scale were used to assess the variation in response within the sample population. The following steps were used to generate a total score for each scale. Firstly, each item of the scale was assigned a number from high to low, based on each scale format. For example, if the scale response format was strongly agree, moderately agree, agree, disagree, moderately disagree and strongly disagree, then the numbers were assigned as 6, 5, 4, 3, 2 and 1. Secondly, negatively worded items were reversed before a total score was calculated for the scales in order to check reliability. Thirdly, 'not applicable' was excluded from the items. Fourthly, treating each of the items as an interval-level measure, the scores for each were added up to give an overall score for each scale.

Two types of missing values required attention in this study. The first involved item non-response, where there was no response to a particular question or item. The second occurred when the answer was marked in a way that was unclear, or fell outside the range of permissible responses for each item. In order to minimise the amount of missing data for each item, a 'best estimation' method was used whereby respondents with missing data were assigned the item-score of a respondent with a similar age, gender, income, and education level [37-39].

Statistical Package for Social Science (SPSS) 12.01[40] was used for PCA and for the calculation of Cronbach's *alpha*.

### Ethics

Ethical approval was obtained from the Human Research Ethics Committee, Queensland University of Technology (QUT) before conducting the above described pre-testing and the following mailed survey (QUT Ref No 3642H).

## Results

The initial PCA produced eleven factor components with eigenvalues above 1 (range: 1.022 -5.352), which explain a total of 68.67 per cent of the variance. Using the screeplot provided by SPSS (not shown), there was quite a clear break between the fifth and sixth components. Components 1 to 5 captured much more of the variance (48.46%) than the remaining components. Therefore, five factor components were determined.

The final PCA rotation then produced five factors that measured social support, perceived interpersonal care, concerns about availability of and accessibility to health care, value of good health, and attitudes towards health care, which explained 12.8%, 10.9%, 10%, 7.9% and 6.8% of the variance, respectively.

Table 3 presents the final component loadings for all of the complete scale items and the retained factor loadings are highlighted in bold. All ten items of the "social support" scale were retained. All three items of the scale "perceived interpersonal care" were retained, which were loaded together with one item (item (a)) from the scale "value of general practitioners" along with the item (c) from the scale "concerns about availability of and accessibility to health care". The new scale was created with five items and was still named "perceived interpersonal care". It should be noted that the response format for the new scale "perceived interpersonal care" was combined with other two items from two different response formats. The final response format suggested for this scale was "Excellent, very good, good, fair and poor". The rest of items (b-e) from the scale "Value of GPs" were removed because they had a factor loading less than 0.5. The scale "concerns about availability of and accessibility to health care" became eight items. All five items of "value of good health" were also retained. Three items (a-c) from "attitudes towards health care" were retained, but item (d) was removed.

**Table 3:** Results of final Principal Component Analysis for the scales

Scales/items		Factors and item loadings (Varimax Rotation)				
		
		1	2	3	4	5
Social support						
a.	People don't visit me as often as I would like.	0.565	-0.163	-0.126	-0.014	0.246
b.	I often need help from other people but can't get it. ^a^	0.662	-0.056	-0.105	-0.065	0.250
c.	I seem to have a lot of friends. ^a^	0.512	0.155	-0.028	0.004	0.026
d.	I don't have anyone that I can really talk to.	0.785	-0.002	-0.076	-0.026	0.213
e.	I have no one to lean on in times of trouble.	0.796	0.020	-0.044	-0.056	0.216
f.	There is someone who can always cheer me up. ^a^	0.701	0.086	0.084	0.118	-0.110
g.	I often feel very lonely.	0.709	-0.085	-0.161	0.014	0.274
h.	I enjoy the time I spend with the people who are important to me. ^a^	0.529	0.168	0.036	0.099	-0.269
i.	When something is on my mind, just talking with the people I know makes me feel better. ^a^	0.510	0.180	0.099	0.183	-0.262
j.	When I need someone to help me, I can usually find someone.	0.745	0.179	0.037	0.105	- 0.202
Perceived interpersonal care						
a.	The amount of time the doctor spends with you?	0.116	0.848	0.028	-0.096	-0.012
b.	The doctor's patience with your questions or worries?	0.116	0.882	-0.041	-0.064	0.028
c.	The doctor's caring and concern for you?	0.080	0.889	-0.038	-0.066	0.020
Concerns about availability of and accessibility to health care						
a.	Being able to find a doctor who bulk bills	-0.174	-0.148	0.520	-0.039	-0.092
b.	Having a number of doctors to choose from	-0.014	0.042	0.577	0.122	-0.056
c.	Being able to see my preferred doctor every time	-0.033	0.523	0.328	0.124	0.049
d.	The amount of time it takes to get an appointment	0.059	0.102	0.662	-0.016	-0.022
e.	The cost of seeing a doctor	-0.163	-0.052	0.681	0.009	-0.098
f.	Transport to see a doctor or medical centre	-0.089	0.113	0.691	0.066	0.094
g.	The amount of time to travel to see a doctor	-0.024	-0.006	0.704	0.083	0.124
h.	The amount of time I have to wait in the waiting room	0.111	-0.094	0.650	-0.088	-0.104
i.	The amount of time I get to spend with a doctor	0.058	0.227	0.626	0.020	0.030
Value of General practitioners						
a.	The care I have received from doctors in the last few years has been good	0.195	0.733	-0.101	-0.004	0.126
b.	Doctors belong to a very high status profession	0.060	0.414	0.022	0.106	0.098
c.	A person understands their own health better than most doctors do	0.061	-0.024	-0.009	-0.091	0.388
d.	It is very important to choose your doctor carefully to get good medical care ^a^	0.057	-0.415	-0.154	-0.177	0.102
e.	Many doctors are more interested in their incomes than in making sure everyone receives adequate medical care	0.212	0.237	-0.242	-0.056	0.342
Value of good health						
a.	Get more rest and sleep	-0.018	0.031	0.009	0.757	0.053
b.	Get more exercise	0.087	0.020	-0.006	0.718	-0.011
c.	Cut down on the amount of work you do	0.042	0.056	-0.019	0.701	0.043
d.	Stop eating some favourite foods	0.003	0.055	0.056	0.714	0.043
e.	Spend more time doing things with family and friends	0.100	0.112	0.120	0.716	-0.070
Attitudes towards health care						
a.	If you wait long enough, you can get over almost any disease without seeing a doctor	0.086	0.052	-0.058	0.105	0.631
b.	I avoid seeing a doctor whenever possible	0.022	0.151	0.067	0.097	0.751
c.	I only go to a doctor if there is no other option	0.010	0.101	0.019	0.068	0.789
d.	Even if a person is feeling okay, they should get a general examination or check up every year or so ^a^	-0.053	0.339	-0.007	0.163	0.063
**Total Variance Explained**		**12.78%**	**10.94%**	**10.01%**	**7.93%**	**6.80%**

Notes: a. The score for this item has been reversely converted from original score, which gave a negative coefficient.

Table 4 presents the results of the internal reliability test for each scale, including mean scores, standard deviations and Cronbach's *Alpha *reliability coefficients. The reliability coefficients for the scales of social support (*alpha *0.86), perceived interpersonal care (*alpha *0.87), concerns about availability of and accessibility to health care (*alpha *0.80), value of good health (*alpha *0.79), and attitudes towards health care (*alpha *0.75) were all acceptable.

**Table 4:** Mean scores, standard deviations and Cronbach's *Alpha *reliability coefficients for the factorially derived scales

Scales	Number of Cases	Number of Items (possible score range)	Mean (SD)	Cronbach's *Alpha*
Social Support	381	10 (10-60)	44.78 (7.64)	0.86
Perceived interpersonal care	378	5 (5-25)	15.02 (3.88)	0.87
Concerns about availability of health care and accessibility to health care	323^a^	8 (8-40)	29.22 (5.65)	0.80
Value of good health	381	5 (5-20)	15.12 (2.51)	0.79
Attitudes towards health care	381	3 (3-18)	11.69 (3.25)	0.75

Notes: a. 'Not applicable' was excluded from the scales.

## Discussion

This research has identified five scales that measure some important factors related to people's use of GP preventive health services in Australia. Scale development and testing were conducted according to six key steps, that included: (1) identifying and conceptualising the domains to be measured; (2) identifying and/or adapting existing scales and additional scales to be developed; (3) revising the scales and items and then constructing the questionnaire and having this reviewed by experts; (4) further pre-testing and refining the scales and pilot questionnaire; (5) collecting data using a population-based survey methodology; and finally, (6) conducting a formal psychometric evaluation of the scales. The six derived scales were formally tested for their validity and reliability. Subsequently, five relevant scales were identified: (1) social support, (2) perceived interpersonal care, (3) concerns about availability of health care and accessibility to health care, (4) value of good health, and (5) attitudes towards health care. Each of the scales was also found to have an acceptable reliability coefficient [35].

There are three specific strengths to this research. Firstly, the research used a theoretical framework derived mainly from Anderson's Health Services Utilisation Model [1], to guide domain and scale identification, scale development and validation. Secondly, the study used a socio-economically diverse and randomly selected population-based sample, so as to ensure the future generalizability of the developed scales. Finally, the study provided evidence of both factorial validity and internal reliability for the scales, which can be used by researchers in future research, particularly in Australia and potentially also, in other countries.

However, there are also a number of methodological shortcomings that need to be considered. The study sample was drawn from the electoral roll in the local government area of Brisbane, using a method which has been previously shown to under-represent socially disadvantaged individuals [41,42]. The results from the representativeness analysis indicated that the respondents with a higher level of education in the study sample were over-represented, whereas respondents with a lower level of education were underrepresented when compared to the 2001 census of the Local Government area of Brisbane. The use of the scales needs to be further evaluated in low socioeconomic individuals or communities. Data were based on self-report which is a commonly used method of collecting data about individuals' health and risk-factor status [43]. Newell et al. [43] critically reviewed 66 studies based on self-reports of health behaviours and risk factors relating to cancer and cardiovascular diseases in the general population. They concluded that self-reported data consistently underestimated the proportion of individuals considered 'at-risk'. Furthermore, the research findings relate primarily to GP-based utilisation of preventive health services by the general adult population in Australia and we do not really know the applicability or relevance of these scales for use in other population with different health care systems in other counties. Future research in different utilisations of different health care systems may be needed, as well as the salient factors for each of the systems.

## Conclusion

The five scales are suitable for further use by researchers and practitioners interested in measuring people's perception, attitudes and beliefs towards GP preventive health services utilisation at individual, interpersonal, organisational/environmental and systems level. Given this study used people's perceptions of these factors, more direct (objective) measures to assess these are necessary in the future. Nevertheless, it is imperative to identify such factors impacting people's use of preventive health services, in order to prevent chronic diseases[44]. It is especially important for those groups where there is poorer access and utilisation of the services [45-47].

## Competing interests

The authors declare that they have no competing interests.

## Authors' contributions

JZ: Made substantial contributions to conception and study design, carried out data collection and performed the statistical analysis and interpretation of data. Has also been involved in drafting the manuscript, revised it critically for important intellectual content and given final approval for the version to be published. BO: Made substantial contributions to conception and study design. Has also been involved in drafting the manuscript, revised it critically for important intellectual content and given final approval for the version to be published. GT: Made substantial contributions to study design, data analysis and interpretation of data. Has been involved in drafting the manuscript, revised it critically for important intellectual content and given final approval for the version to be published.

## Pre-publication history

The pre-publication history for this paper can be accessed here:

http://www.biomedcentral.com/1472-6963/9/218/prepub
